# Medical Items

**Published:** 1913-04

**Authors:** 


### MEDICAL ITEMS

### "DAMAGED GOODS"

The presentation of Brieux's "Damaged Goods" as announced in our last month's issue took place according to arrangement on the afternoon of March 14th. The audience—one of the most intellectual and cultured ever seen in New York City—showed a keen interest in the problem unfolded by the group of players Richard Bennett had induced to give the play. As is pretty generally known Brieux's famous play depicts the dangers of syphilis, especially to the innocent. It is not necessary to outline the story, but suffice it to say, that the author with consummate skill emphasizes the evils of ignorance, the dire results of which so often occur solely because, as the Doctor says, "they didn't know."

Those who were fortunate in getting seats saw a finished performance that reflected great credit on every one taking part. Wilton Lackaye as the Doctor and Richard Bennett as the young husband who disobeyed the doctor's mandate that he postpone his marriage for several years, never appeared to better advantage. Indeed it is doubtful if these splendid actors have ever done any finer work in their lives than the portrayal of these two leading characters of Brieux's play. No one who saw them will soon forget the finesse with which each played his part. Quite as much commendation can be given to the rest of the cast and it is rare indeed that a group of players presenting such uniform ability and talent is brought together. Throughout the play there was in evidence an earnestness and sincerity, that plainly reflected a general recognition by every member of the cast of the epoch making character of the undertaking.

Without the slightest desire to exaggerate it can be said that no recent event of sociologic importance has had more far-reaching possibilities along practical lines than the presentation of "Damaged Goods.'

It augurs well for the morale of the stage when our leading actors and actresses are willing at great personal sacrifice to give their time and effort to the promulgation of truth and the promotion of ideals. The educated and cultured people of New York owe a real debt to Richard Bennett and his associates for this courage, zeal and broad humanitarianism. That their efforts were appreciated has been pretty conclusively indicated by the fact that two overflow performances have had to be given to accommodate those who were unable to get seats at the first presentation. It is also significant that the play will be reproduced in Washington as a result of a special invitation from certain Washington people who recognized its tremendous possibilities in connection with urgently needed legislation. In every respect the enterprise reflects great credit on all who have aided in any way to make the reproduction of this remarkedly play, a sociologic as well as a dramatic success. We are especially proud that an American medical journal should have been an important factor in this splendid undertaking and contributed so much to its gratifying outcome. We extend to the Medical Review of Reviews our heartiest congratulations for its splendid work and the success that has crowned its unselfish efforts.—American Medicine.

